# 

**Published:** 1890-03-22

**Authors:** 


### NEW OFFICES IN THE STRAND.

The Hospital *is now published at* **140, Strand, W.C.** *It has been found desirable to establish* The Hospital *in this central and convenient locality, owing to the rapid extension of the business of this paper of late.*

### NOTICE TO CORRESPONDENTS.

All MS., Letters, Books for Review, and other matters intended for the Editor should be addressed The Editor, The Lodge, Porchester Square, London, W.

**Notice to Advertisers and Subscribers.**

All Advertisements, Orders for Copies of The Hospital, and Business Communications should be addressed to The Publisher, *not to the Editor*, The Hospital, 140, Strand, W.C.

**Bound Volumes of "The Hospital."**

Vol. VI., containing full page portraits of T.R.H. the Prince and Princess of Wales, and Miss Florence Nightingale, is now ready, 4s. post free. Cases for binding may be had of the Publisher, at 1s. 6d. each.

**To Residents, Secretaries, and Matrons.**

The Editor will be glad to have the Regulations and each Report as issued by Asylums, Hospitals, Convalescent and Nursing Institutions, as well as those of other Charities, and an early copy in duplicate of all Appeals and Festival Notices, etc., addressed to *The Editor, The Lodge, Porchester Square, London, W.* Any superintendent, secretary, matron, or other chief official who regularly sends such information may be registered, on application to the Publisher, to receive free of cost a cover for binding The Hospital in half-yearly volumes.

### Scraps and Gleanings.

Influenza is prevalent in Cairo.

Shrewsbury Eye, Ear, and Throat Hospital treated 340 in-patients last year.

Last Saturday's *Athenæum* contained on article on "Thomas Guy as a Publisher."

The new Sidcup Cottage Hospital has been commenced; Mr. Sydney G. Goss is architect.

Sir Richard Owen has recovered from his late illness, and no further bulletins will be issued.

The Bread and Food Reform League are organizing dinners for children attending the London Board Schools.

A comprehensive temperance movement is being organised by the Roman Catholic authorities in Ireland.

The death is announced of Miss Sewell, the first M.B. in America, and a constant and welcome visitor to London.

There is no medical department of the Jewish Board of Guardians, neither have the Board any medical officers.

At Lisbon a death from trichinosis has taken place, and the authorities are instituting rigorous precautionary measures.

The Queen has given a donation of £10 towards the funds of the Winchester Diocesan Mission to the Deaf and Dumb.

The patients of the Cancer Hospital were lately cheered by an excellent amateur performance of "Camaralzaman."

The Princess of Wales on the 8th inst. granted an interview to Miss MacCulloch, Resident Medical Officer to the New Hospital for Women.

The Paris Academy of Medicine has just opened to competition a prize of the value of 1000 francs for the best work on the hygiene of infants.

On the 6th inst., Mrs. S. Halford, as in former years, treated the patients in the Jewish Convalescent Home, South Norwood, to a Purim dinner.

Dr. Edward Berdoe, author of "St. Bernards" is bringing out a book on "Browning's Message to His Times." The subject of vivisection is introduced.

The Governor of Hong-Kong has been pleased to appoint Mr. J. Cantlie, M.B., and Mr. J. M. Atkinson, M.B., to be Justices of the Peace for that colony.

The influenza epidemic seems to be well-established in India, accounts of its prevalence coming from Bombay, Poonah, Jubbulpore, Benares, Lucknow, and elsewhere.

The London Temperance Hospital has received £5,000 on account of its share of residue under a trust deed executed by the late Mr. George Sturge, of Sydenham-hill.

The Duke of Westminster has arranged to preside at a drawing-room meeting in Grosvenor House, on Friday afternoon, April 25th, on behalf of the Ragged School Union.

During last month the officers of the Fishmongers' Company seized at Billingsgate 106 tons of fish as unfit for human food, of which no less than 103 tons were Norway herrings.

The medical officer to the Kidderminster Town Council reports that there had been 5,000 cases of influenza in the borough, but no death has been registered from the disease.

The Animals' Institute in Kinnerton Street, Wilton Place, is doing a good work in the interests of the animal world, and last week held an exhibition of new inventions in horse shoes.

The Phillips Memorial Hospital at Bromley, issues its first annual report; 18 in-patients were treated last year, and a large number of visits were made to patients in their own homes.

The influenza has been making great ravages in Teheran, where several members of the Royal family have caught the illness, and 70 deaths have been registered in the city on one day.

The Lord-Lieutenant of Ireland has conferred the honour of knighthood upon Dr. John Nugent, who has just retired from the Chief Inspectorship of Lunatic Asylums in Ireland, after forty-three years' service.

Miss Jane Burnup, of Barros Bridge, Newcastle, a lady who was widely known for her great benevolence and philanthropy, and who died on February 13th, has by her will bequeathed £2,100 to various local charities.

Dr. Hay, of Bridport, and his coachman were driving from Chidcock, near Bridport, at midnight, when the horse ran into a hedge, throwing both violently out of the trap. The coachman—whose neck was broken—died instantly.

The Tenth International Medical Congress will be held in Berlin from August 4th to August 9th. The work of the Congress will be discharged by 18 different sections. The official languages of all the sittings will be German, English, and French.

The treasurer of the Royal Southern Hospital, Liverpool, has received from the executors of the late Isaac Denton a legacy of £1,000, bequeathed by him to that institution. Mr. Denton had been for many years a generous supporter of the hospital.

The Examiners of Water state that during the month of February they took 168 samples of water from the mains of the seven London water companies, and that, with the exception of two samples, the whole were clear, bright, and well-filtered.

Several cases of a new disease originally appeared in Mantua immediately after the subsidence of the recent influenza epidemic, and to which the people of that city gave the name of "Na nonna" (*anglice*, falling asleep), have occurred in the comitat of Pressburg. People suffering from this complaint fall into a death-like trance lasting about four days, out of which the patient wakes in a state of intense exhaustion.

The medical officer of health for the Cheshunt Urban Sanitary Authority reports that the death-rate in the district for the past year was equal to 10.6 per thousand of the population—a rate which will compare favourably with many seaside resorts. The Cheshunt Local Board has expended within the past two or three years something like £61,000 in works of drainage, water supply, and general improvement.

